# *Begonia wuzhishanensis* (sect. *Diploclinium*, Begoniaceae), a new species from Hainan Island, China

**DOI:** 10.1186/1999-3110-55-24

**Published:** 2014-02-05

**Authors:** Ching-I Peng, Xiao-Hua Jin, Shin-Ming Ku, Yoshiko Kono, Han-Yau Huang, Hsun-An Yang

**Affiliations:** 1 Herbarium (HAST), Biodiversity Research Center, Academia Sinica, Nangang, Taipei 115 Taiwan; 2 State Key Laboratory of Systematic and Evolutionary Botany & Herbarium, Institute of Botany, Chinese Academy of Sciences, Beijing, 100093 China; 3 School of Forestry and Resource Conservation, National Taiwan University, Daan, Taipei 106 Taiwan

**Keywords:** *Begonia wuzhishanensis*, Begoniaceae, Chromosome number, Flora of China, Hainan, New species, Sect. *Diploclinium*, Septal placentation

## Abstract

**Background:**

Hainan is the largest island of the Indo-Burma Biodiversity Hotspot and has the best preserved and most extensive tropical forests in China. A recent study on distribution of endangered species in China identifies southern Hainan as one of eight hotspots for plant conservation in the country. In continuation of our studies of Asian *Begonia*, we report the discovery of an attractive undescribed species, *B. wuzhishanensis* C.-I Peng, X.H. Jin & S.M. Ku, from Hainan Island.

**Results:**

Living plant of the new species, *Begonia wuzhishanensis,* was collected in 2009 and cultivated in the experimental greenhouse for morphological and cytological studies. It flowered consecutively in 2012 and 2013 in the experimental greenhouse, Academia Sinica. It was assigned to the large, heterogeneous sect. *Diploclinium*. The chromosome number of this new species was determined to be 2*n* = 26.

**Conclusions:**

A careful study of literature, herbarium specimens and living plants, both in the wild and in cultivation, support the recognition of the new species *Begonia wuzhishanensis*, which is described in this paper. *Begonia wuzhishanensis* is currently known only from Fanyang, Wuzhishan Mountain in the center of the island. A line drawing, color plate, and a distribution map are provided to aid in identification.

**Electronic supplementary material:**

The online version of this article (doi:10.1186/1999-3110-55-24) contains supplementary material, which is available to authorized users.

## Background

Hainan is the largest island of the Indo-Burma Biodiversity Hotspot and has the best preserved and most extensive tropical forests in China (Deng et al. [2008]; Zang and Ding [2009]). The island harbors the Hainan Island Monsoon Rain Forest Ecoregion, one of the 26 terrestrial habitats of China internationally recognized by the World Wildlife Fund for its global ecological importance (Francisco-Ortega et al. [2010]). A recent study on distribution of endangered species in China identifies southern Hainan as one of eight hotspots for plant conservation in the country (Zhang and Ma [2008]). In Hainan eight species of *Begonia*, four of which endemic (*B. hainanensis* Chun & F. Chun, *B. howii* Merr. & Chun, *B. peltatifolia* H.L. Li, and B*. sublongipes* Y.M. Shui) to the island, were documented (Gu et al. [2007]). In continuation of our studies of Asian *Begonia* (e.g., Chung et al., [2014]; Hughes et al. [2011]; Nakamura et al. [2013]; Peng et al. [2012][2013][2014]; Rubite et al.: *Begonia chingipengii* [sect. *Baryandra*, Begoniaceae], a new species from Luzon Island, Philippines, submitted), we report the discovery of an additional undescribed species, *B. wuzhishanensis*, endemic to the island.

## Methods

### Chromosome preparations

Somatic chromosomes of the new species, *Begonia wuzhishanensis* (*Ku & Jin 2093*) were examined using root tips. The methods of pretreatment, fixation and staining for chromosome observations followed Peng et al. ([2012]). Classification of the chromosome complements based on centromere position at mitotic metaphase follows Levan et al. ([1964]). Voucher specimens have been deposited in Herbarium, Biodiversity Research Center, Academia Sinica, Taipei (HAST).

### Cryo scanning electron microscopy

A fresh leaf of *Begonia wuzhishanensis* (*Ku & Jin 2093*) was dissected and attached to a stub. The samples were frozen with liquid nitrogen slush, then transferred to a sample preparation chamber at −160°C and etched for 15 min at −85°C. After etching, the temperature reached −130°C for sample fracturing and coating. After coating, the samples were transferred to the SEM chamber and observed at −160°C with a cryo scanning electron microscope (FEI Quanta 200 SEM/Quorum Cryo System PP2000TR FEI).

## Results and discussion

### Species description

**Figure 1:**
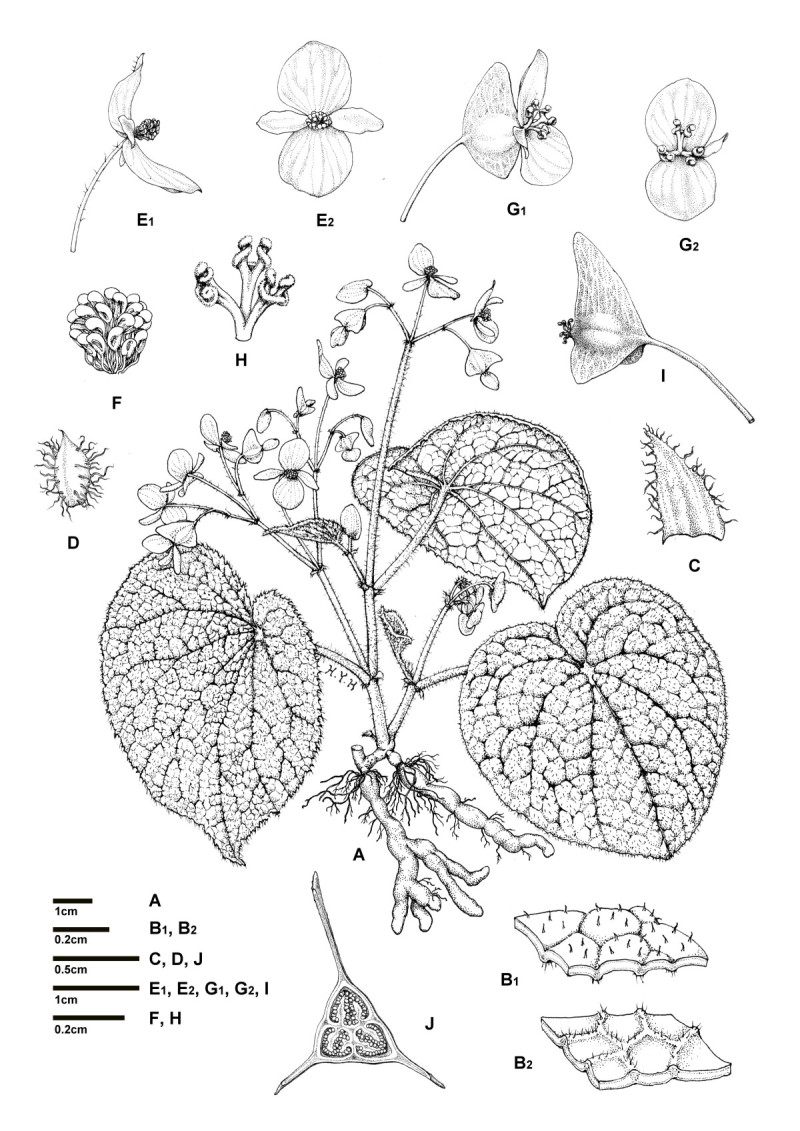
***Begonia wuzhishanensis*** **C.-I Peng, X.H. Jin & S.M. Ku. A**, Habit; **B1**, Leaf adaxial surface; **B2**, Leaf abaxial surface; **C**, Stipule; **D**, Bract; **E1**, Staminate flower, side view **E2**, Staminate flower, face view; **F**, Androecium; **G1**, Carpellate flower, side view; **G2**, Carpellate flower, face view; **H**, Style and stigmas; **I**, Capsule. All from *Ku & Jin 2093* (HAST).

**Figure 2:**
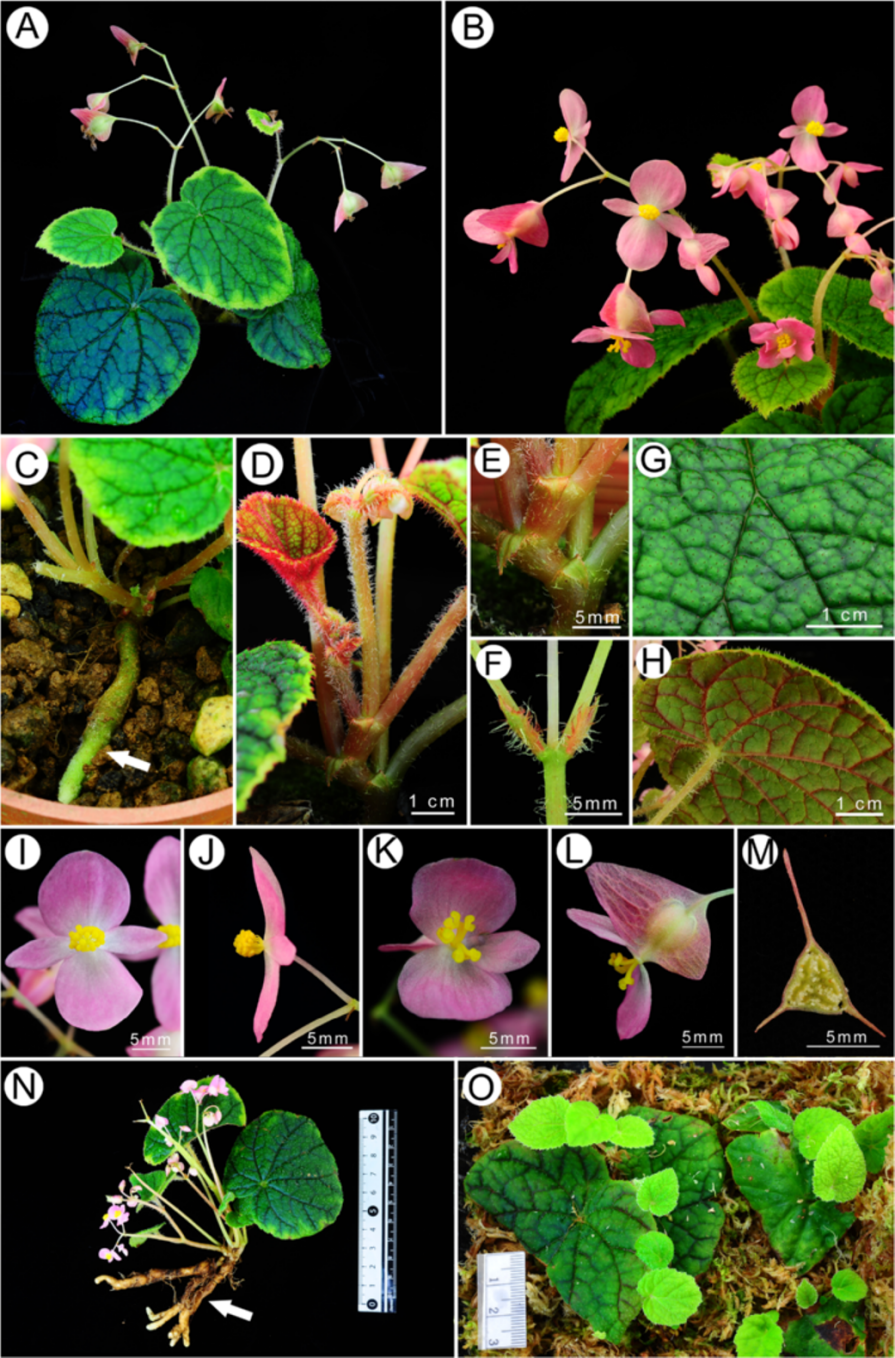
***Begonia wuzhishanensis*** **C.-I Peng, X.H. Jin & S.M. Ku. A**, Habit, leaf showing iridescence; **B**, Inflorescence; **C**, Elongate tuber (arrow); **D**, Erect stem; **E**, Stipules; **F**, Bracts; **G**, Leaf adaxial surface; **H**, Leaf abaxial surface; **I**, Male flower, face view; **J**, Male flower, side view; **K**, Female flower, face view; **L**, Female flower, side view; **M**, Cross-section of ovary; **N**, Plant with subterranean, branched, elongate tubers (arrow); **O**, Vegetative propagation. All from *Ku & Jin 2093* (HAST).

*Begonia wuzhishanensis* C.-I Peng, X.H. Jin & S.M. Ku, sp. nov. (sect. *Diploclinium*) —TYPE: CHINA, Hainan Province, Wuzhishan City, Fanyang Township, Nanyi Village, on wet, mossy rocky slope by a stream in forest, elev. ca. 180 m, 18°52'53"N, 109°20'33"E, 10 October 2009. Type specimens pressed from plants brought back from the field and cultivated in the experimental greenhouse, Academia Sinica, Taiwan, 3 December 2013. *Shin-Ming Ku & Xiao-Hua Jin 2093* (holotype: HAST; isotype: PE). 五指山秋海棠 Figures 1 and 2.

Herbs, monoecious, deciduous perennial, with elongate tubers. *Tubers* prostrate on soil surface or underground, to 7 cm long, 0.9 cm across, branched or unbranched. *Stems* erect, succulent, 0.3–0.5 cm across, to 20 (−25) cm high at anthesis, branched from lower nodes. *Leaves* alternate, petioles 2.5–4 cm long, 0.4 cm across; stipules triangular, margin fimbriate; leaf blade nearly symmetric, cordate, 3.5–8 cm long, 3–7.5 cm wide, chartaceous, adaxial surface green, slightly bullate, hirtellous, venation dark green and impressed, iridescent; leaf abaxial surface pale, veins reddish or red, manifestly elevated and hirtellous, margin irregularly double-serrate. *Inflorescences* axillary, dichasial cymes branched 2–3 times, peduncle 10–18 cm long, ca. 0.3 cm in diameter, hirtellous; lower bracts ovate, ca. 0.5 cm long, 0.3 cm wide, margin fimbriate, abaxially densely hirtellous, eventually deciduous. *Staminate flower:* pedicel sparingly hirtellous to nearly glabrous, 2–4 cm long, tepals 4, pinkish, outer 2 broadly ovate, ca. 1 cm long, 0.8 cm wide, abaxially glabrous to hirtellous, inner 2 lanceolate, ca. 0.8 cm long, 0.4 cm wide, glabrous; androecium zygomorphic, ca. 0.3 cm across; stamens ca. 25–30; filaments free, 0.1–0.2 cm long; anthers 2-locular, obovoid. *Carpellate flower*: pedicel sparingly hirtellous to subglabrous, 1.3–1.6 cm long, tepals 3, outer 2 broadly ovate to suborbicular, ca. 0.8 cm long, 0.8 cm wide, inner 1 lanceolate, ca. 0.6 cm long, 0.4 cm wide; ovary trigonous-ellipsoid, ca. 0.7 cm long, 0.5 cm across, white, glabrous, 3-winged, 3-locular; placentas septal; styles 3. *Capsule* nodding, ca. 1.6 cm long, 1.2 cm wide, wings unequal, triangular, abaxial wing ca. 1.2 cm tall, lateral wings ca. 0.4 cm tall, styles persistent.

#### Additional specimens examined

CHINA. Hainan Province, Wuzhishan City, Fanyang, in forest, elev. ca. 200–500 m, 15 October 2003, *Xiao-Hua Jin 5208*; ca. 300–500 m, 20 October 2003, *Xiao-Hua Jin 5284* (PE).

#### Chromosome cytology

**Figure 3:**
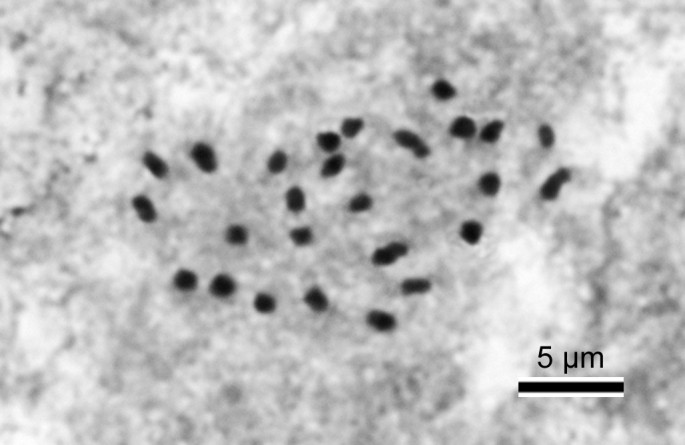
**Somatic chromosomes at metaphase of** ***B. wuzhishanensis*** **(2** ***n*** **= 26, from** ***Ku & Jin 2093*** **, HAST).**

Somatic chromosomes at metaphase of *B. wuzhishanensis* (*Ku & Jin 2093*, HAST) were determined to be 2*n* = 26 (Figure 3). The 26 chromosomes gradually varied from ca. 1.0 to 2.0 μm long in length. A pair of the longest chromosomes has centromeres at submedian; several chromosomes at median, however, those of smaller chromosomes could not be determined. Satellites were not observed.

*Begonia wuzhishanensis* belongs to the heterogeneous section *Diploclinium,* which comprises ca. 130 species (Doorenbos et al. [1998]; Hughes and Pullan [2007]). To our knowledge, 16 species of the section were cytologically studied. They exhibited a wide variation of chromosome numbers as 2*n* = 22, 24, 26, 28, 30, 32, 36, 38, 46, 52, 82. Somatic chromosome number of 2*n* = 26 was previously reported in two species: *B. acaulis* Merr. & L.M. Perry (Papua New Guinea, Legro and Doorenbos [1969]) and *B. grandis* Dryand. (China and Japan, Nakata et al. [2012]).

#### Leaf anatomy and vestiture

**Figure 4:**
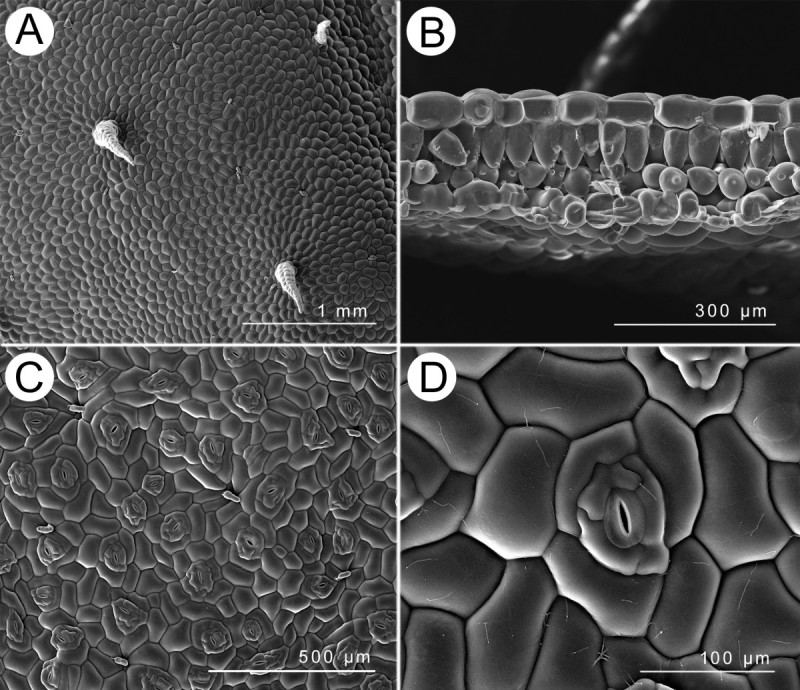
**Leaf SEM microphotographs of** ***B. wuzhishanensis*** **. A**, Adaxial surfaces; **B**, Cross section; **C**, Abaxial surfaces; **D**, Stomata complex.

Adaxial surface with scarce multiseriate trichomes (Figure 4A); cross section ca. 220 μm thick, epidermis single-layered on both surfaces, hypodermis absent, palisade tissue and spongy tissue both single-layered (Figure 4B); abaxial surface with multiseriate trichomes on nerves, stomata complex single, helicocytic, nearly flat (Figure 4C,D).

#### Ecology and distribution

**Figure 5:**
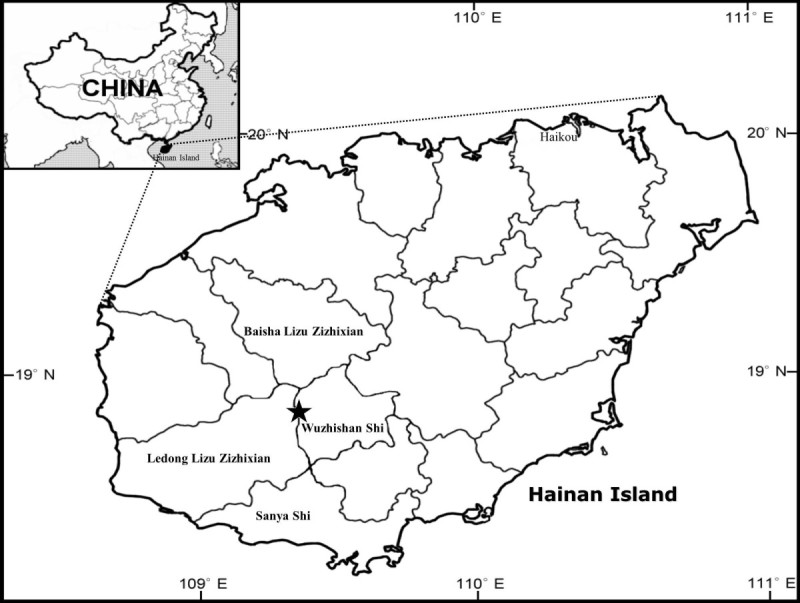
**Distribution of** ***Begonia wuzhishanensis*** **(★) in Hainan Island.**

Wuzhishan (1,876 m), located in the center of Hainan, is the highest mountain on the island. *Begonia wuzhishanensis* is a rare species found at lower part (ca. 200–500 m altitude) of Wuzhishan (Figure 5). It occurs on wet mossy rocks by streams in tropical forest.

#### Etymology

The specific epithet is derived from the type locality, Wuzhishan ('Five-fingered Mountain', literally) of Hainan Island, China.

#### Phenology

Flowering from August to December; fruiting from October to December.

#### Cultivation

*Begonia wuzhishanensis* can be grown from seeds. Alternatively, they can be propagated by the elongate tubers or leaf cuttings with ease (Figure 2O).

#### Notes

**Figure 6:**
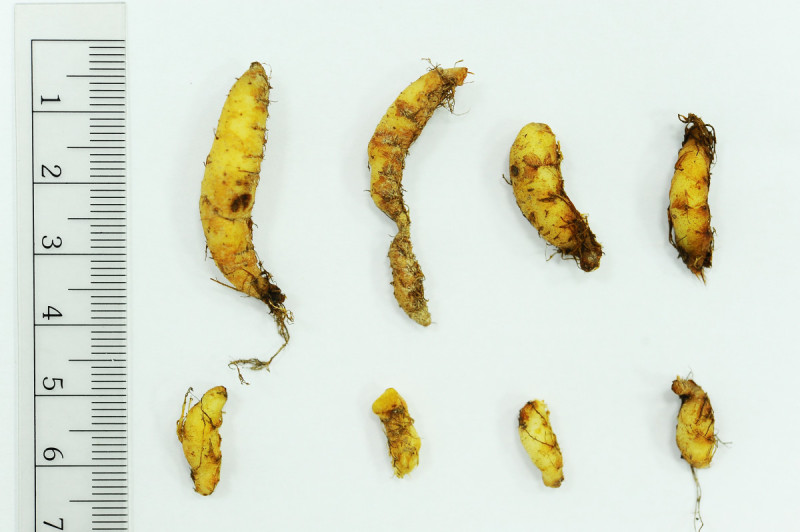
**Elongate tubers of** ***Begonia woodii*** **Merr. (Philippines, Palawan, Taytay, Lake Danao,** ***Peng 23479*** **, HAST).**

*Begonia wuzhishanensis* has prominent elongate tubers that are rarely encountered in the genus. This feature resembles those observed in *B. lithophila* C.Y. Wu (sect. *Reichenheimia*; Li [2006]) from Yunnan, China and *B. woodii* Merr. (sect. *Baryandra*) from Palawan, the Philippines (Figure 6). The perennating tubers of *B. woodii* have been erroneously interpreted as short rhizomes in the past (Merrill [1925]; Hughes et al. [2010]).

**Figure 7:**
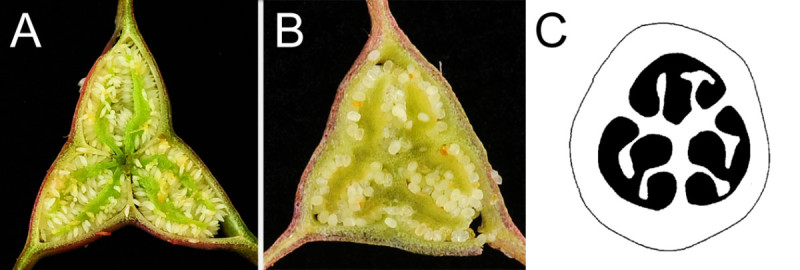
**Placentations. A**. Typical axile placentation (*Begonia ravenii* C.-I Peng & Y.K. Chen, sect. *Diploclinium*); **B**. Septal placentation (*B. wuzhishanensis*); **C**. Schematic drawing of septal placentation (adopted from Reitsma, [1984]: Figure 1D).

Placentation of *B. wuzhishanensis* is noteworthy: its ovary is 3-locular with 2 placentas each growing from middle of the septa (Figures 1J, 2M, 7B). Such placentation was classified as 'septal' in African *Begonia* (Figure 7; Reitsma [1984]). Septal placentation was also observed in part of the ovary of some species in the Asian sect. *Coelocentrum* (e.g. *B. aurantiflora*; C.-I Peng, Yan Liu & S.M. Ku, Peng et al. [2008]: Figure one; *B. bamaensis*; Yan Liu & C.-I Peng, Liu et al. [2007]: Figure one; *B. ×breviscapa*;C.-I Peng, Yan Liu & S.M. Ku, Peng et al. [2010]: Figure one; *B. debaoensis*;C.-I Peng, Yan Liu & S.M. Ku, Ku et al. [2006]: Figure one; *B. semiparietalis*;Yan Liu, S.M. Ku & C.-I Peng, Ku et al. [2006]: Figure nine). With 3-locular ovaries, bifid placentas and perennating tubers, etc. but without axile placentation in *B. wuzhishanenis* (Figure 7B), we have tentatively placed it in the polymorphic and 'dust-bin' sect. *Diploclinium* (Rubite et al. [2013]). Further molecular phylogenetic studies to clarify its placement are underway.

*Begonia wuzhishanensis* somewhat resembles *B. obsolescens* Irmsch. and *B. fimbristipula* Hance. Comparison of salient features of the three species is provided in Table 1.

**Table 1:** **Comparison of** ***Begonia wuzhishanensis*** **,** ***B. fimbristipula*** **and** ***B. obsolescens***

	***B. wuzhishanensis***	***B. fimbristipula***	***B. obsolescens***
Perennating by	Elongate tubers	Subglobose Tubers	Rhizomes
Leaf shape	Nearly symmetric	Nearly symmetric	Asymmetric
Tepal number in carpellate flower	3	3	5
Placentation	Septal	Axile	Axile

## Conclusions

A careful study of literature, herbarium specimens and living plants, both in the wild and in cultivation, support the recognition of the new species *Begonia wuzhishanensis*. It is currently known only from Fanyang, Wuzhishan Mountain in the center of Hainan island, China. *Begonia wuzhishanensis* is unique in having elongate tubers and septal placentation. It is tentatively assigned to sect. *Diploclinium*. Further studies to clarify its phylogenetic position are underway.

## Authors’ original submitted files for images

Below are the links to the authors’ original submitted files for images. Authors’ original file for figure 1 Authors’ original file for figure 2 Authors’ original file for figure 3 Authors’ original file for figure 4 Authors’ original file for figure 5 Authors’ original file for figure 6 Authors’ original file for figure 7

## References

[CR1] Chung K-F, Leong W-C, Rubite RR, Repin R, Liu Y, Peng C-I (2014). Phylogenetic analyses of Begonia sect. *Coelocentrum* and allied limestone species of China shed light on the evolution of Sino-Vietnamese karst flora. Bot Stud.

[CR2] Deng F, Zang R, Chen B (2008). Identification of functional groups in an old-growth tropical montane rain forest on Hainan Island, China. Forest Ecol Manag.

[CR3] Doorenbos J, Sosef MSM, de Wilde JJFE (1998). The sections of Begonia including descriptions keys and species lists. Studies in Begoniaceae VI.

[CR4] Francisco-Ortega J, Wang Z-S, Wang F-G, Xing F-W, Liu H, Xu H, Xu W-X, Luo Y-B, Song S-Q, Gale S, Boufford DE, Maunder M, An S-Q (2010). Seed plant endemism on Hainan Island: a framework for conservation actions. Bot Rev.

[CR5] Gu C, Peng C-I, Turland NJ, Wu Z-Y, Raven PH, Hong D-Y (2007). Begoniaceae. Flora of China, vol 13.

[CR6] Hughes M, Coyle C, Rubite RR (2010). A revision of *Begonia* section *Diploclinium* (Begoniaceae) on the Philippine Island of Palawan, including five new species. Edinburgh J Bot.

[CR7] Hughes M, Pullan M (2007). Southeast Asian Begonia.

[CR8] Hughes M, Rubite RR, Kono Y, Peng C-I (2011). *Begonia blancii* (sect. *Diploclinium*, Begoniaceae), a new species endemic to the Philippine island of Palawan. Bot Stud.

[CR9] Ku S-M, Liu Y, Peng C-I (2006). Four new species of *Begonia* sect. *Coelocentrum* (Begoniaceae) from limestone areas in Guangxi, China. Bot Stud.

[CR10] Legro RAH, Doorenbos J (1969). Chromosome numbers in *Begonia* 1. Netherlands J Agric Sci.

[CR11] Levan A, Fredga K, Sandberg AA (1964). Nomenclature for centromeric position on chromosomes. Hereditas.

[CR12] Li H-Z (2006). Studies on conservation biology of Begonia sect. Reichenheimia in China. Ph. D. dissertation.

[CR13] Liu Y, Ku S-M, Peng C-I (2007). *Begonia bamaensis* (sect. *Coelocentrum*, Begoniaceae), a new species from limestone areas in Guangxi, China. Bot Stud.

[CR14] Merrill ED (1925). Additions to our knowledge of the Philippine flora I. Philipp. J. Sci.

[CR15] Nakamura K, Rubite RR, Kono Y, Callado JR, Peng C-I (2013). *Begonia tandangii* (Begoniaceae, section *Baryandra*), a new species from Luzon Island, the Philippines. Phytotaxa.

[CR16] Nakata M, Ueno T, Li J-X, Li H-Z, Wang Z-L, Lu Y-X, Shen Y-G, Guan K-Y (2012). Chromosome number and pollen fertility of *Begonia grandis* (Begoniaceae) from Japan and China. Bull Bot Gard Toyama.

[CR17] Peng C-I, Liu Y, Ku S-M (2008). *Begonia aurantiflora* (sect. *Coelocentrum*, Begoniaceae), a new species from limestone areas in Guangxi, China. Bot Stud.

[CR18] Peng C-I, Liu Y, Ku S-M, Kono Y, Chung K-F (2010). *Begonia* × *breviscapa* (Begoniaceae), a new intersectional natural hybrid from limestone areas in Guangxi, China. Bot Stud.

[CR19] Peng C-I, Ku S-M, Kono Y, Liu Y (2012). *Begonia chongzuoensis* (sect. *Coelocentrum*, Begoniaceae), a new calciphile from Guangxi, China. Bot Stud.

[CR20] Peng C-I, Yang H-A, Kono Y, Chung K-F, Huang Y-S, Wu W-H, Liu Y (2013). Novelties in *Begonia* sect. *Coelocentrum*: *B. longgangensis* and *B. ferox* from limestone areas in Guangxi, China. Bot Stud.

[CR21] Peng C-I, Wang H, Kono Y, Yang H-A (2014). *Begonia wui-senioris* (sect. *Platycentrum*, Begoniaceae), a new species from Myanmar. Bot Stud.

[CR22] Reitsma JM (1984). Placentation in Begonias from the African continent. Meded. Landbouwhogeschool Wageningen.

[CR23] Rubite RR, Hughes M, Alejandro GJD, Peng C-I (2013). Recircumscription of *Begonia* sect. *Baryandra* (Begoniaceae): evidence from molecular data. Bot Stud.

[CR24] Zang R, Ding Y (2009). Forest recovery on abandoned logging roads in a tropical montane rain forest of Hainan Island, China. Acta Oecol.

[CR25] Zhang Y-B, Ma K-P (2008). Geographic distribution patterns and status assessment of threatened plants in China. Biodivers Conserv.

