# Supplementary figures and images for: Begonia wuzhishanensis (sect. Diploclinium, Begoniaceae), a new species from Hainan Island, China
Source: Bot Stud. 2014 Feb 5;55:24. doi: 10.1186/1999-3110-55-24 (PMC5430360; doi:10.1186/1999-3110-55-24)

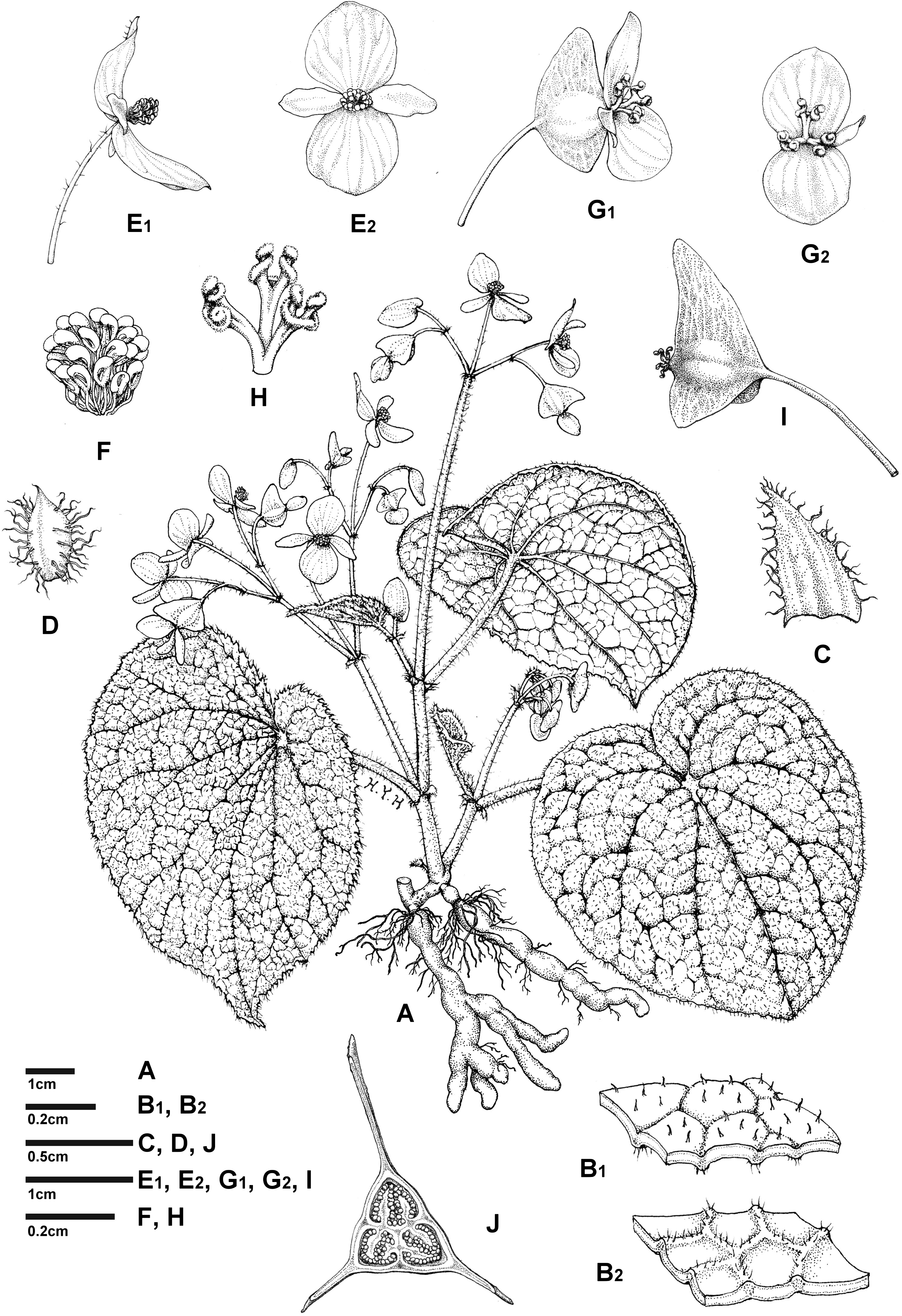

Supplement: Supplementary file 1 — Authors’ original file for figure 1 [file 40529_2013_72_MOESM1_ESM.tif]

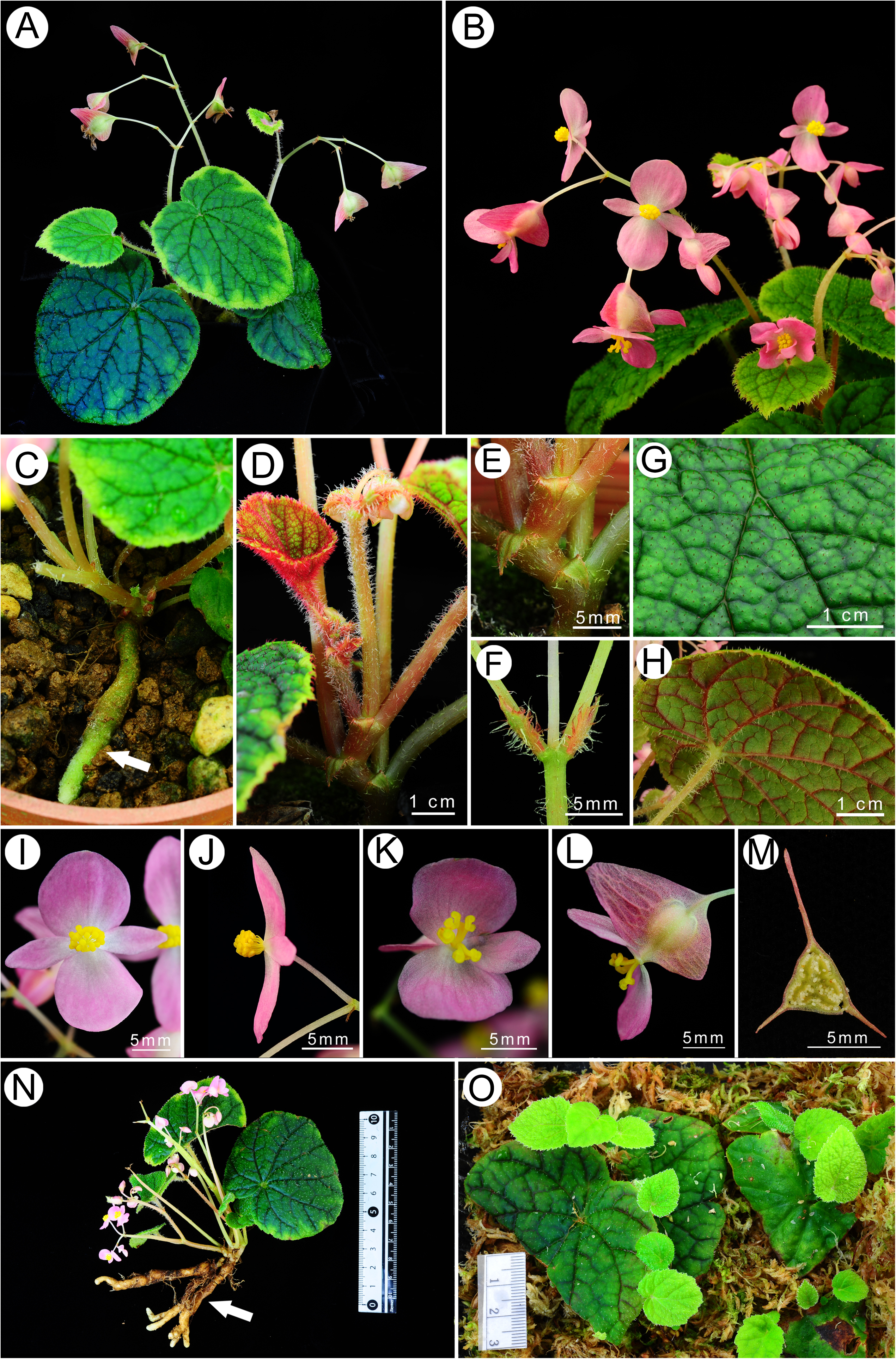

Supplement: Supplementary file 2 — Authors’ original file for figure 2 [file 40529_2013_72_MOESM2_ESM.tif]

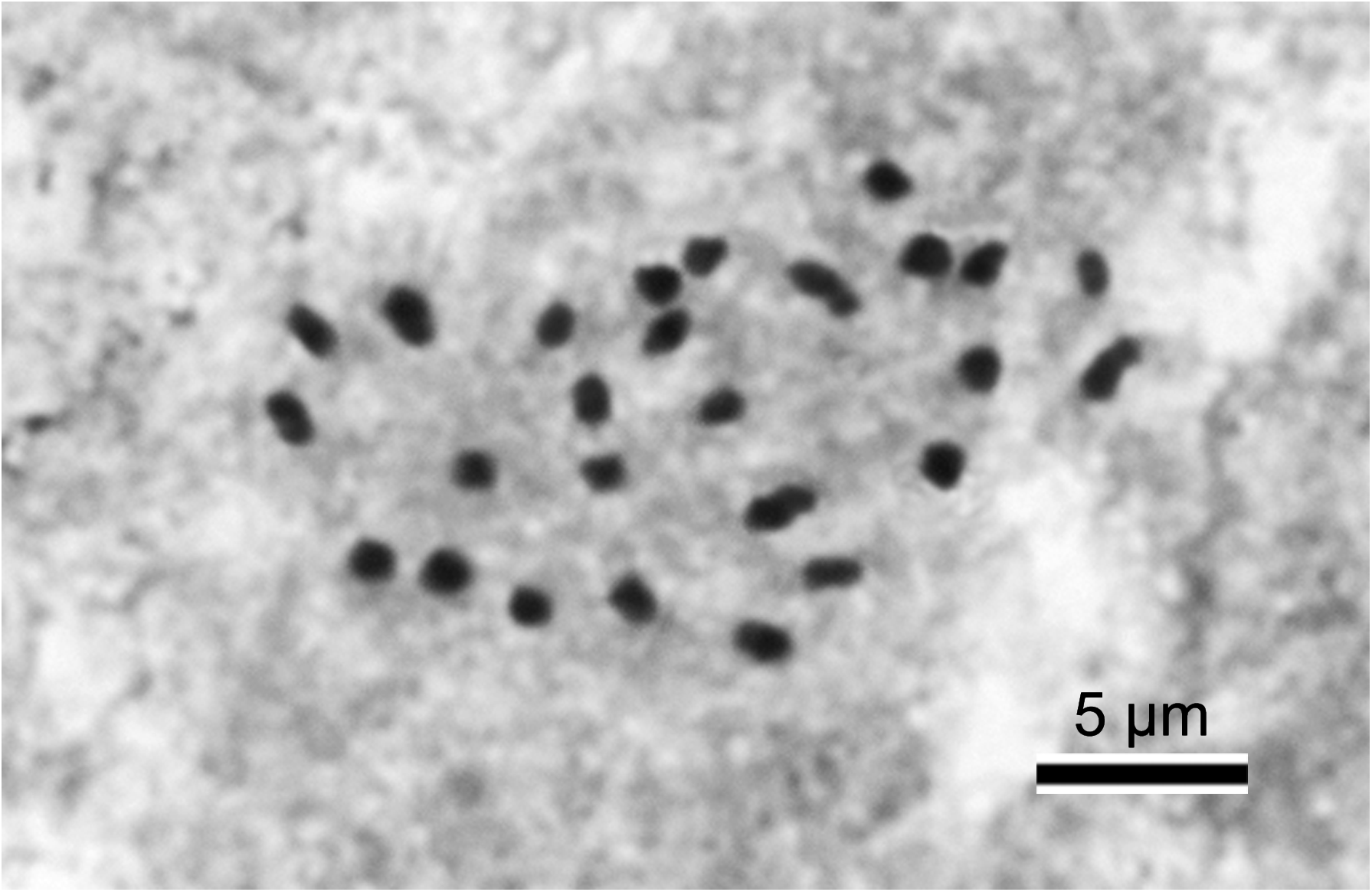

Supplement: Supplementary file 3 — Authors’ original file for figure 3 [file 40529_2013_72_MOESM3_ESM.tif]

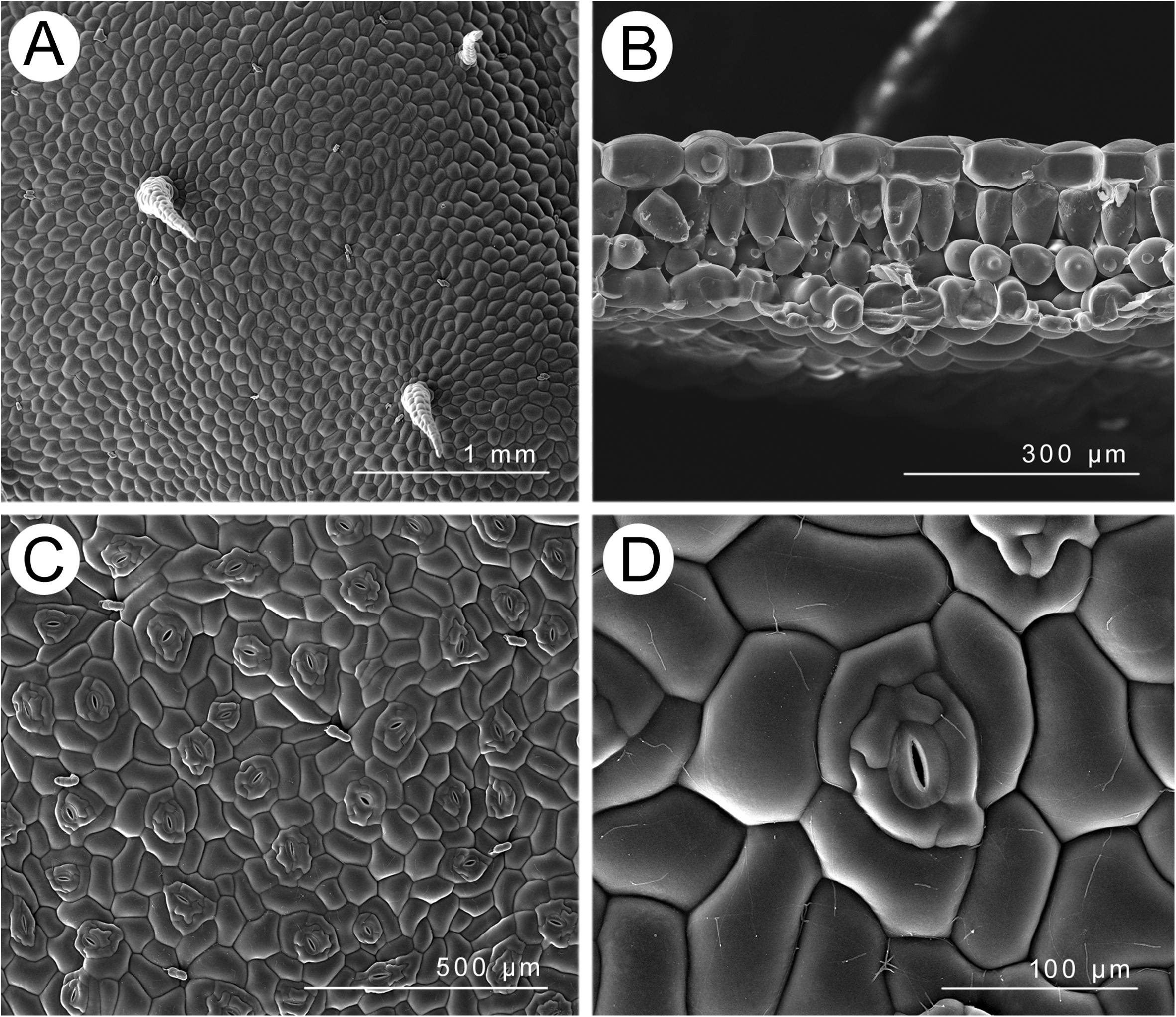

Supplement: Supplementary file 4 — Authors’ original file for figure 4 [file 40529_2013_72_MOESM4_ESM.tif]

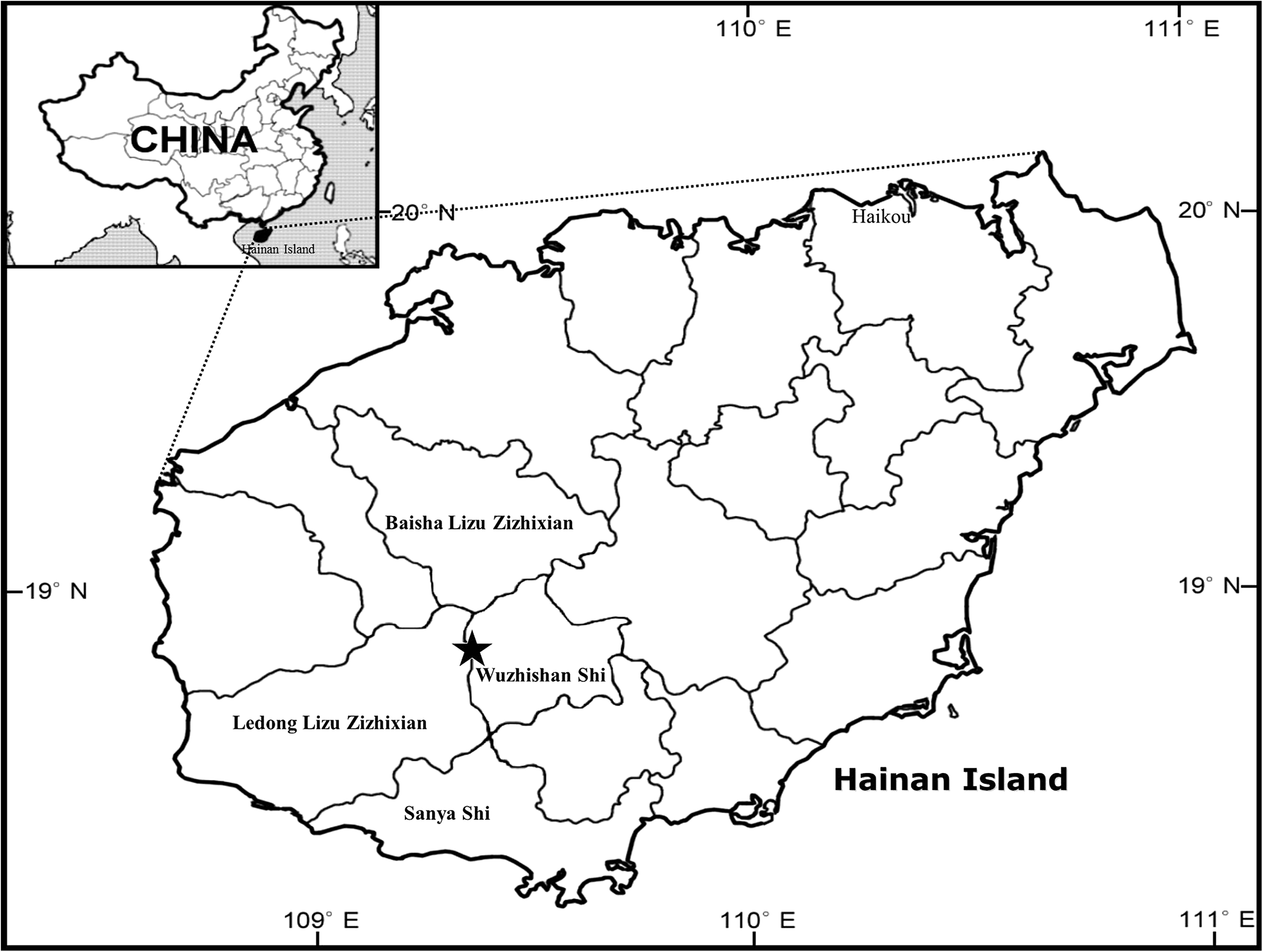

Supplement: Supplementary file 5 — Authors’ original file for figure 5 [file 40529_2013_72_MOESM5_ESM.tif]

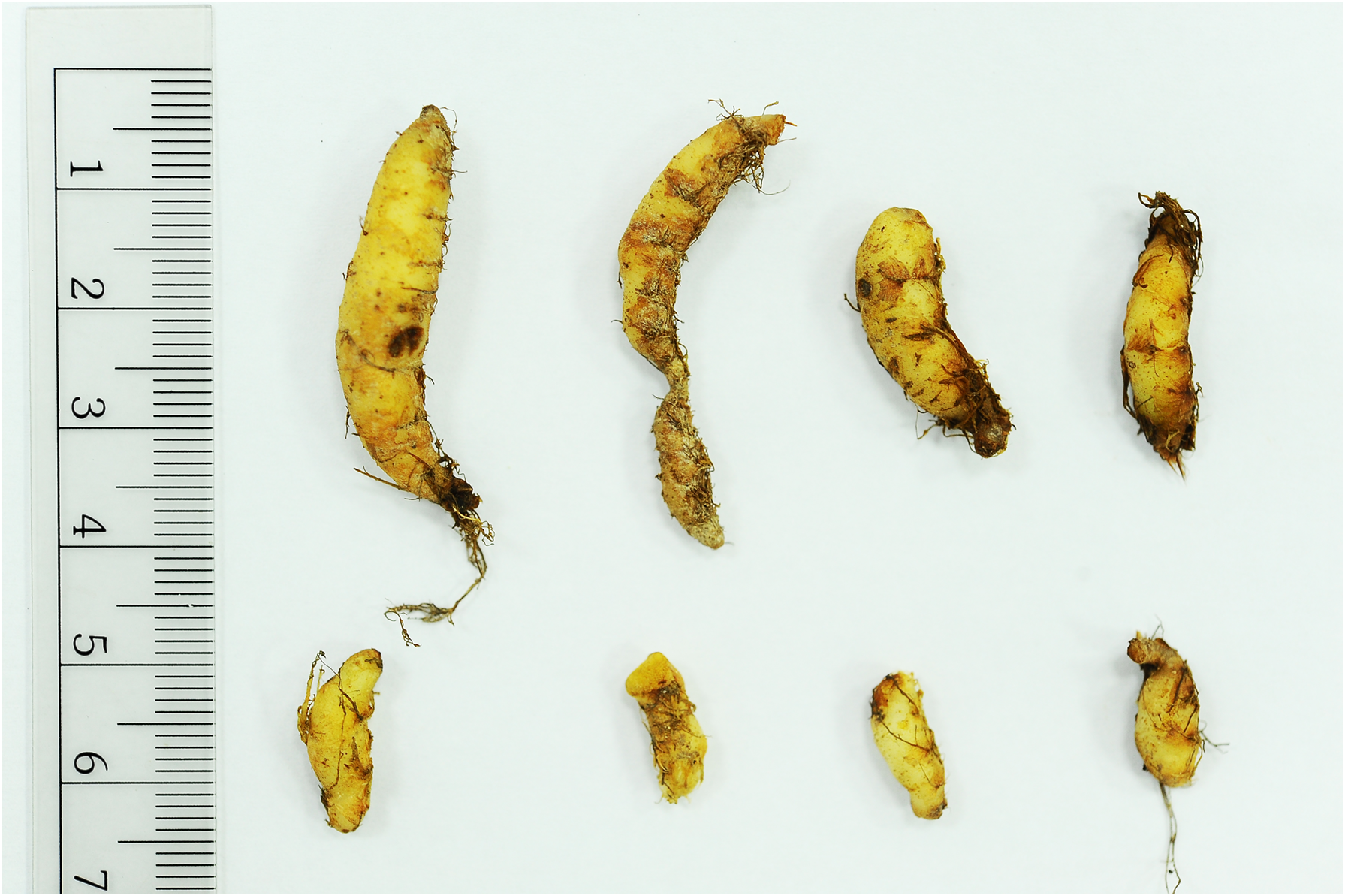

Supplement: Supplementary file 6 — Authors’ original file for figure 6 [file 40529_2013_72_MOESM6_ESM.tif]

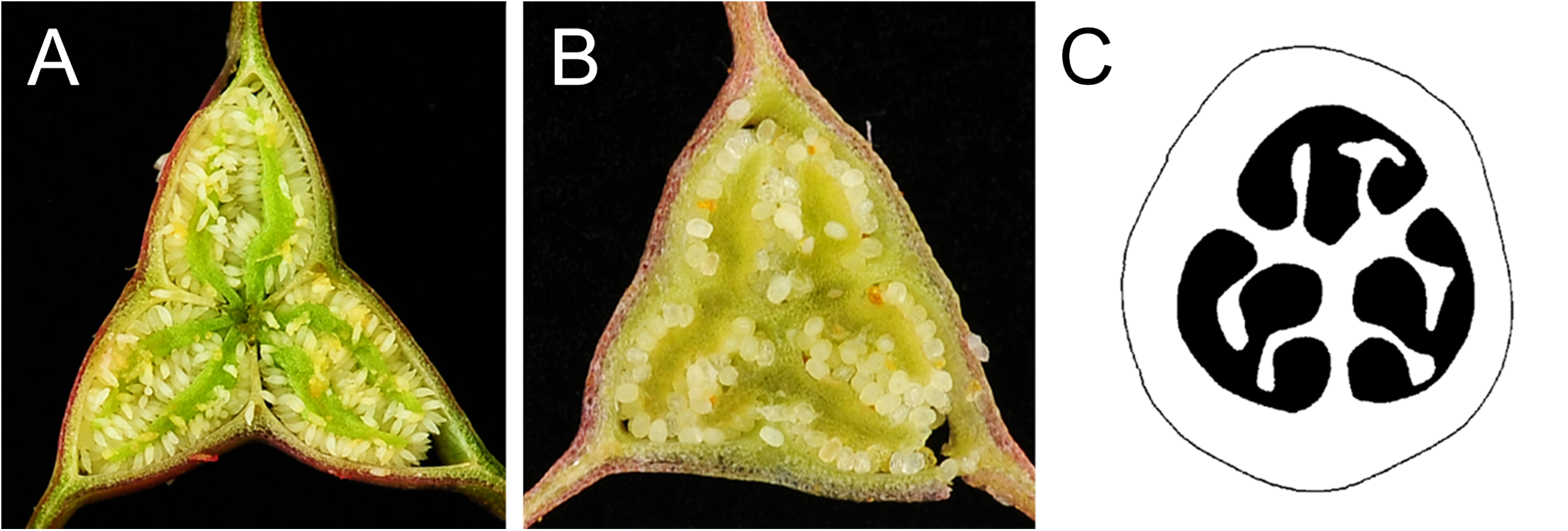

Supplement: Supplementary file 7 — Authors’ original file for figure 7 [file 40529_2013_72_MOESM7_ESM.tif]
